# Spread of a highly mucoid *Streptococcus pyogenes emm*3/ST15 clone

**DOI:** 10.1186/1471-2334-10-233

**Published:** 2010-08-05

**Authors:** Esther Tamayo, Milagrosa Montes, Guadalupe García-Medina, José M García-Arenzana, Emilio Pérez-Trallero

**Affiliations:** 1 Biomedical Research Centre Network for Respiratory Diseases (CIBERES), San Sebastián, Spain; 2 Microbiology Service, Hospital Donostia, San Sebastián, Spain; 3 Department of Preventive Medicine and Public Health, Faculty of Medicine, Basque Country University, San Sebastián, Spain

## Abstract

**Background:**

Hyaluronic acid capsule plays a key role in *Streptococcus pyogenes *virulence. Circulation of mucoid or highly encapsulated strains has been related to rheumatic fever epidemics and invasive disease in several countries. In 2009, an outbreak of mucoid *S. pyogenes *isolates was detected in northern Spain. The aim of the study was to describe clinical and molecular characteristics of mucoid strains causing this outbreak and to compare them with a sample of non-mucoid *S. pyogenes *isolates obtained during the same period of time.

**Methods:**

All *S. pyogenes *isolates with a mucoid colony morphology (n = 132), 10% of non-mucoid (n = 144) and all invasive *S. pyogenes *isolates (n = 7) obtained in 2009 were included. Characterization was performed by T-agglutination, *emm *typing, pulsed field gel electrophoresis and multilocus sequence typing.

**Results:**

One clone characterized as *emm*3.1/ST15 comprised 98.5% (n = 130) of all mucoid isolates. Subjects of all ages were affected. Main clinical manifestations were pharyngitis and scarlet fever, but this clone also caused invasive disease: two cases of streptococcal toxic shock syndrome, one arthritis, and one celullitis with a fatal outcome. Mucoid isolates were more prone to cause invasive disease than non-mucoid isolates (p = 0.001).

**Conclusions:**

Although no acute rheumatic fever cases were detected, the most worrisome characteristics of this clone were the success for causing invasive disease and the merge of two virulent features: the serotype, *emm*3, and capsule hyper-production, expressed as a mucoid morphology.

## Background

Group A streptococcus (GAS) strains differ widely in their encapsulation degree, and those with an exuberant capsule production have a mucoid morphology when are cultured on blood agar plates. The expression of the capsule has been related to the virulence of the strain since it was first recognized [1,2]. In the United States temporal association between a high incidence of rheumatic fever cases and an increased presence of mucoid *S. pyogenes *strains was observed [3]. More concretely, the rheumatogenic ability has been associated to a limited number of *emm *types such as *emm*1, 3, 5, 6, or 18 which often were mucoid [1,3] although this pattern is not held in all parts of the world, like in Hawaii [4]. Furthermore, these *emm *types, and particularly *emm*1 and *emm*3 types, are the most prevalent *emm *types found among specimens causing invasive GAS disease worldwide [5,6]. By performing blood agar cultures for *S. pyogenes *detection, phenotypic characteristics like mucoid colony morphology can be recognized. In fact, recently the advisability of detecting mucoid isolates in clinical practice has been emphasized in the literature [1].

In 2009, a sudden emergence of encapsulated *S. pyogenes *isolates was observed among clinical samples received at the microbiology laboratory of Hospital Donostia, (Gipuzkoa), in Northern Spain, where circulation of mucoid *S. pyogenes *strains was highly infrequent in the past. The aim of this work was to describe molecular characteristics of mucoid isolates causing this outbreak and to compare them to a sample of non-mucoid *S. pyogenes *isolates obtained in the same period of time.

## Methods

### Geographical location

The province of Gipuzkoa is situated in the north of Spain, flanked by France in the north. Hospital Donostia, in Gipuzkoa, is the referral hospital for the province, and attends a population of 405,745 inhabitants of all ages, children included. The microbiology laboratory receives all throat samples from Primary Care Centers of the area to investigate the presence of GAS.

### Isolate sample and identification

All *S. pyogenes *isolates with a mucoid colony morphology (n = 132) detected in 2009 were included in the study. Also, randomly selected 10% of non-mucoid *S. pyogenes *isolates from pharyngeal swabs (n = 144) and all *S. pyogenes *isolates from sterile body sites (n = 7) obtained that year were analyzed. To investigate the presence of GAS, samples were routinely cultured on blood agar plates and incubated overnight in an atmosphere enriched with 5% of CO_2 _at 37°C. Colonies were identified as GAS by bacitracin-susceptibility and agglutination with specific Group-A streptococci antisera (Slidex Strepto-kit; bioMérieux, Marcy l'Etoile, France).

### Isolate characterization

Characterization was performed by T-agglutination, *emm *typing, pulsed field gel electrophoresis (PFGE) and multilocus sequence typing (MLST) as described elsewhere [7]. T-type was done by agglutination of trypsin digested bacteria suspensions using commercial poly- and mono-specific T antisera according to manufacturer's instructions (Sevapharma, Prague, Czech Republic). *emm*-type was determined by polymerase chain reaction-restriction fragment length polimorfism (PCR-RFLP) assay. In those isolates were *emm *gene did not amplify, specific recommendations with a lysate preparation for mucoid strains were followed (available at: http://www.cdc.gov/ncidod/biotech/strep/protocol_emm-type.htm). The *emm*-gene of all invasive isolates, and at least 15% of each different *emm*-type of non-invasive (mucoid and non-mucoid) isolates were sequenced according to the guidelines of the Division of Bacterial and Mycotic Diseases, Centers for Disease Control and Prevention (CDC) (available at: http://www.cdc.gov/ncidod/biotech/strep/M-ProteinGene_typing.htm). All mucoid isolates (n = 132) and all non-mucoid *emm*3 (n = 24) isolates were investigated by PFGE. Samples were digested with *Sma*I enzyme, and results were analyzed by the Diversity Database fingerprinting software, version 2 (BioRad, USA) to construct a dendrogram by the unweighted pair group method with arithmetic averages, Dice coefficient, and a position tolerance of 1%. Isolates belonging to the same PFGE pattern had a similarity higher than 85%. A sample representing at least 10% of each PFGE pattern (n = 18 isolates) was characterized by MLST (available at: http://spyogenes.mlst.net/misc/info.asp).

### Antibiotic susceptibility testing

Erythromycin, clindamycin, tetracycline and levofloxacin susceptibility testing was performed by broth microdilution method, using Sensititre microtitre trays (Trek Diagnostic Systems, East Sussex, UK) with Mueller-Hinton II broth (BioMerieux, Mercy l'Etoile, France) supplemented with lysed horse blood (3-5% vol/vol). Minimum inhibitory concentrations (MICs) were performed and interpreted according to the criteria recommended by the Clinical and Laboratory Standards Institute (CLSI) [8].

### Statistical analysis

Data were analyzed with the Instat3 program. Chi square and Fisher's exact probability tests were used to perform comparisons.

### Ethics Statement

In the present study no human experimentation was conducted, with all studies carried out on microorganisms. The study and publication of their results was approved by the 'Comité Ético de Investigación Clínica del Área Sanitaria de Gipuzkoa'.

## Results and Discussion

**Figure 1:**
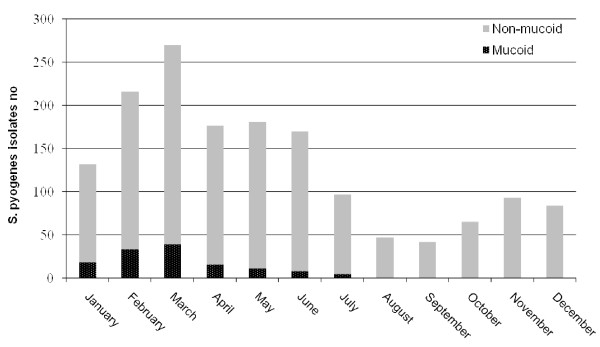
**Circulation of mucoid and non-mucoid *S. pyogenes *isolates in 2009 (n = 1571)**.

In 2009, 1571 *S. pyogenes *isolates were detected among samples received at the microbiology laboratory of Hospital Donostia. Of them, 132 were mucoid, representing the 8.4% of all isolates of that year, while in the previous four years only 9 mucoid isolates were detected. Circulation of mucoid isolates through 2009 is represented in figure 1.

**Figure 2:**
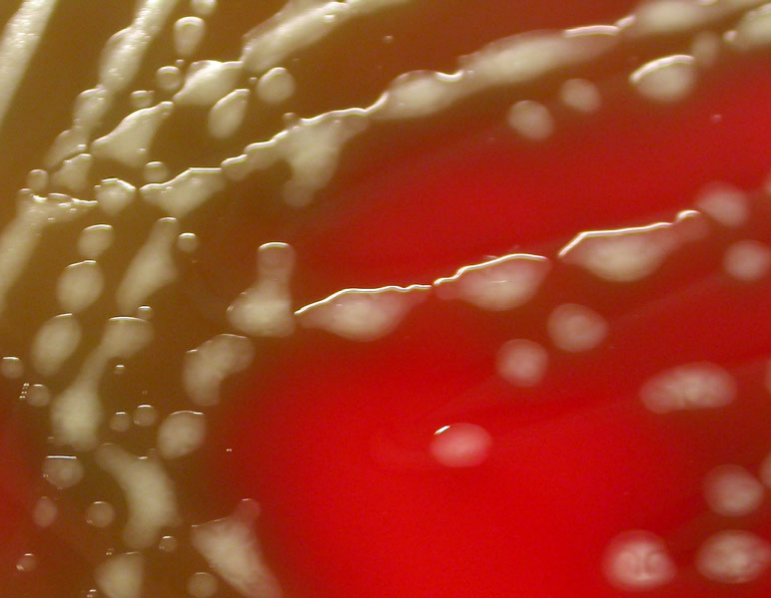
**Mucoid *S. pyogenes *colonies on blood agar plates after overnight incubation under an atmosphere enriched with 5% CO_2_**.

Crater *et al. *[9] demonstrated that capsule production is greatest in the early logarithmic growth phase in broth cultures and that its expression is lost when the stationary growth phase is reached. In our experience, mucoid colonies showed a wet and glossy shape in blood agar plates (figure 2), although few of them were already dried showing a colapsed, rough and "matte" morphology different to that found in non-mucoid colonies. Incubation of blood agar plates in an atmosphere enriched with 5% of CO_2 _enhanced capsule expression. All mucoid isolates were susceptible to erythromycin, clindamycin, tetracycline and levofloxacin antimicrobials showing the following MICs: erythromycin MIC < 0.5 μg/mL; clindamycin MIC < 0.5 μg/mL; tetracycline MIC < 4 μg/mL; and levofloxacin MIC < 2 μg/mL.

**Figure 3:**
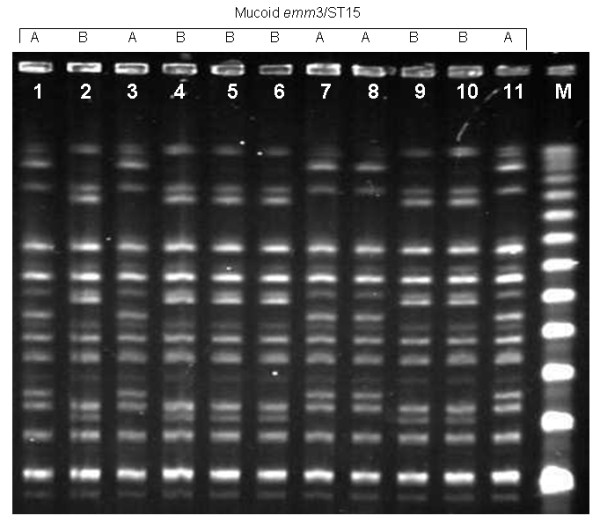
**PFGE patterns found among *emm*3/ST15 mucoid *S. pyogenes *isolates: lanes1, 3, 7, 8 and 11 represent pattern A; lanes 2, 4-6, 9 and 10 represent pattern B; lane M, lambda ladder**.

Overall, 98.5% of all mucoid isolates (130/132) belonged to the *emm*3.1 type. The two isolates with a different *emm *type were characterized as *emm*5.46 and *emm*1.0. In a high percentage of mucoid isolates the *emm *gene was difficult to amplify. Probably the vast amount of hyaluronic acid could have interfered in DNA extraction as after following specific CDC recommendations for mucoid strains, a complete *emm *gene amplification success was obtained. PFGE revealed 4 different patterns: patterns A and B, with a similarity of 73% between them for *emm*3 mucoid isolates (figure 3), pattern C for the *emm*1 isolate and pattern D for the *emm*5 isolate. All *emm*3 mucoid isolates, independently of the PFGE pattern, belonged to the ST15, the *emm*1 isolate to the ST28 and the *emm*5 isolate to the ST99. All *emm*3 isolates were non T-typeable. This finding was unrelated to the mucoid capsule because among 89 non-mucoid *emm*3 isolates obtained in the last 4 years, only 1 could be T-typed as T3 (data not shown). The circulation of a mucoid *S. pyogenes *clone (*emm*3.1/ST15) in 2009 contrasts with the results obtained in the past, when only a few mucoid isolates representing a varied sample of *emm *and sequence types were detected in the community.

Twenty four out of 144 non-mucoid isolates analyzed in 2009, belonged to the *emm*3 type, which shared the same molecular and susceptibility characteristics (*emm*3.1/ST15) as the *emm*3 mucoid isolates, although belonged only to the PFGE pattern A. The remaining 120 non-*emm*3 non-mucoid isolates showed percentages of non-susceptibility in variable proportions (19.2% to erythromycin, 5.8% to clindamycin, 5.8% to tetracycline and 1.7% to levofloxacin). The only difference between *emm*3 non-mucoid and mucoid isolates was the degree of hyaluronate encapsulation.

Among mucoid isolates, 87.9% (n = 116) were obtained from throat samples. Age distribution of patients affected by the mucoid clone was similar to that among patients infected by non-mucoid *S. pyogenes *isolates: 84.1% (n = 111) and 80.2% (n = 1154) were children under 14 years old respectively (p = 0.30). The main clinical manifestations were pharyngitis (n = 55), scarlet fever (n = 30) and otitis (n = 13), similar to those found among non-mucoid isolates (n = 1439), although they also caused 4 cases of invasive disease. The *emm*3 type has been ranked among the most prevalent *emm*-types isolated from patients with severe disease worldwide and has been related to high case fatality rates [5,6]. In this study four *emm*3 mucoid isolates were obtained from sterile body sites (3 from blood and 1 from pleural fluid), two of them associated with streptococcal toxic shock syndrome, 1 with arthritis, and 1 with celullitis with a fatal outcome. Among non-mucoid isolates (n = 1439), only 3 cases of invasive disease were registered (one *emm*3.1, one *emm*75, and one *emm*87). These data revealed that invasive disease was more often caused due to mucoid isolates (3%) compared to non-mucoid isolates (0.21%, p = 0.001) in 2009 in our hospital. Independently of the mucoid feature, the *emm*3 type was detected in 71.4% of all invasive GAS disease of that year.

We have found few reports investigating mucoid *S. pyogenes *colony phenotype in Spain. Bosch *et al. *[10] reported 11 out of 31 invasive *S. pyogenes *isolates obtained between 1995 and 1998 showing a mucoid appearance, although only three were heavily mucoid. Sierra *et al. *[11] investigated the presence of mucoid isolates among invasive GAS infections in injecting drug users in Barcelona, finding that all isolates were non-mucoid. By contrast, in other parts of the world, like in the United States, mucoid GAS circulation has been more frequently reported. Veasy *et al. *[3] found an association between mucoid strains circulation and an increase of acute rheumathic fever incidence. Rheumatic fever is considered a rare disease in Europe [12] and no cases were produced in our region in the last ten years. Nevertheless, after the characterization of this clone which merges a traditionally virulent serotype (*emm*3) and the mucoid feature, pediatricians and other physicians were alerted in order to prevent the re-emergence of rheumatic fever into an area previously free of this disease. We have investigated the epidemiology of *S. pyogenes *in our community since the 90's, and to our knowledge *emm*3 *S. pyogenes *isolates circulate every year but without showing a mucoid appearance (data not shown). Why these *emm*3 *S. pyogenes *isolates express the mucoid phenotype is under research.

## Conclusions

To our knowledge, this is the first outbreak of mucoid *S. pyogenes *isolates described in the community in southern Europe, and reminds us of the importance of performing a comprehensive phenotypic and genotypic characterization of circulating strains. It is of special concern that the same isolate merged two important virulence factors: the *emm*-type, *emm*3, and the hiperproduction of capsule observed as a mucoid morphology, which could have enhanced the ability to cause severe disease.

## Competing interests

The authors declare that they have no competing interests.

## Authors' contributions

EPT designed and coordinated the experiments. ET, MM, GGM, JMGA performed the experiments, sequence analysis and analyzed the data. EPT, MM and ET drafted the manuscript. All authors read and approved the final manuscript.

## Pre-publication history

The pre-publication history for this paper can be accessed here:

http://www.biomedcentral.com/1471-2334/10/233/prepub
